# PhysB: a framework for designing conversational agents to support physical activity in adolescents

**DOI:** 10.1186/s41043-026-01254-5

**Published:** 2026-06-06

**Authors:** Richard Moore, Abdel‑Karim Al‑Tamimi, Lambros Lazuras

**Affiliations:** 1 Sheffield Hallam University, Sheffield, UK; 2 University of Lincoln, Lincoln, UK

**Keywords:** Conversational AI, Conversational agents, Cognitive systems, Physical activity, Barriers, Large Language Models, Adolescents

## Abstract

Conversational Agents (CAs) are a promising tool for enhancing adolescents’ physical activity behaviours. To effectively fulfil their role, these agents must address the diverse range of barriers adolescents encounter and provide effective, evidence-based solutions. To achieve this, a theoretical framework titled PhysB is proposed, which is grounded in theory and evidence, allowing for the identification and categorisation of barriers through natural language input. The PhysB framework outlines the relationship between the process of identifying barriers and the cognitive system driving the CA’s interaction with adolescents. It incorporates key constructs from various theoretical models and frameworks and draws from behaviour change theory. The PhysB framework’s main function is to inform the design and evaluation of personalised, evidence-based conversational AI interventions to promote positive behaviour change in adolescents. The PhysB framework’s broad applicability extends to practitioners, policymakers, researchers, and designers involved in designing conversational AI interventions.

## Introduction

A substantial body of literature has emerged in recent years that supports the notion that regular engagement in physical activity (PA) can have a positive impact on multiple aspects of adolescent health and development. Notably, research has established that PA contributes to improved physical and emotional wellbeing [1], enhanced cognitive function [2], and higher academic achievement [3, 4]. Maintaining a consistent level of PA during adolescence may foster healthy habits that extend into adulthood, thereby reducing the risk of inactivity in later years [5–8]. Despite these benefits, a substantial proportion of adolescents worldwide do not engage in enough PA. The 2022 Eurobarometer, which involved 25,578 participants in 27 European countries showed that 36% of people aged between 15–24 years old reported that they seldom or never exercised or played sport [9]. This is alarming given that physical inactivity is a leading cause of premature and preventable mortality, with about 3.2 million deaths attributed to insufficient levels of PA engagement [10]. The WHO (2023) recognises mobile and wireless technology-based solutions as key to effectively promoting PA participation in the population [11]. Accordingly, digital interventions can be effectively used to promote PA participation and reduce sedentary lifestyles and their effects on physical and mental health [12–14].

### Barriers to PA

It is widely understood that multiple barriers to PA exist. Previous research has explored the multitude of barriers which prevent adolescents from participating in PA, from the perspective of adolescents [15–18] and PE (Physical Education) teachers [26]. To design effective behaviour change interventions, it is important to firstly identify the factors that prevent young people from participating in PA. Systematic reviews of qualitative research on this topic have shown that adolescents identify a diverse range of barriers to PA participation. These can be grouped as individual-level barriers (e.g., negative attitudes to PA, lack of motivation, reduced self-efficacy, and body-image issues); social barriers (e.g., social norms among peers and the family; influence from physical education teachers); and institutional and environmental barriers (e.g., access to and opportunities for PA participation; perceived safety of PA facilities) [15–18]. Some of these barriers, such as attitudes to PA, self-efficacy, availability, accessibility, and perceived safety of PA facilities, are also identified in studies among populations with physical and intellectual disabilities [19, 20] and among youth with chronic conditions. Furthermore, physical inactivity levels tend to be higher among female than male youth and among young people at greater risk of socio-economic exclusion (e.g., those from low-income families) [21, 22].

There is consensus that little progress has been made in supporting adolescents to overcome these barriers, necessitating that novel interventions are required to support adolescents [14]. As a result, the Global Action Plan on PA was introduced in 2018 by the World Health Organization (WHO), to stimulate novel interventions aimed at facilitating adolescents’ adherence to the recommended levels of PA [23].

Such novel digital solutions include CAs that have emerged as promising self-help tools to promote and provide support to increase or maintain PA levels [24, 25]. CAs are scalable, cost-effective and accessible any time of the day, serving as a potentially novel and engaging tool for young people to access personalised assistance in overcoming barriers to PA [26]. Consequently, this makes them an appealing option for schools with limited time, resources, and capacity to provide support to adolescents [27]. A further strength of these solutions lies in their capacity to engage in humanlike automated natural language interactions that are personalised to adolescent needs. However, despite their promise, a lack of consistent measurement remains as well as evidence regarding their efficacy [25]. There is a significant gap in research on how cognitive systems — computational systems designed to emulate aspects of human cognition, including learning, reasoning, problem-solving, and natural language understanding [18], can be optimally designed to support this specific purpose and audience, thereby enhancing the effectiveness of CA.

The development of a robust theoretical framework is essential to effectively utilise CAs in addressing barriers to PA among adolescents [18]. Establishing a strong theoretical foundation is required to improve understanding of the underlying mechanisms and factors, creating cognitive systems that contribute to the success of such interventions. The integration of behavioural theories will provide insights into the cognitive and motivational processes that drive behaviour change, facilitating the development of tailored and personalised interventions that address individual barriers and promote sustained engagement in PA [28]. This article aims to address the following research questions:

1. What are the key constructs and theories within the proposed PhysB framework, and how do these constructs relate to each other in the context of promoting PA?
2. How can the PhysB framework guide the design and development of a CA that effectively addresses barriers to PA in adolescents?
3. What are the potential challenges and limitations associated with implementing the PhysB framework as a cognitive system, and how can they be addressed?
4. How does the PhysB framework compare to existing frameworks in terms of its applicability and potential for improving PA outcomes in adolescents?

## The PhysB framework

Innovative strategies are essential to support adolescents in overcoming barriers to PA [14, 23]. CAs offer great promise as a tool to provide personalised support to adolescents [24, 25]. To fulfil their role effectively, these agents must be capable of addressing the intricate array of barriers that adolescents encounter [15–18]. A theoretical framework plays an important role in providing a broad structure and systematic approach to designing and developing effective CAs aimed at supporting adolescents to overcome barriers to PA.

**Fig. 1:**
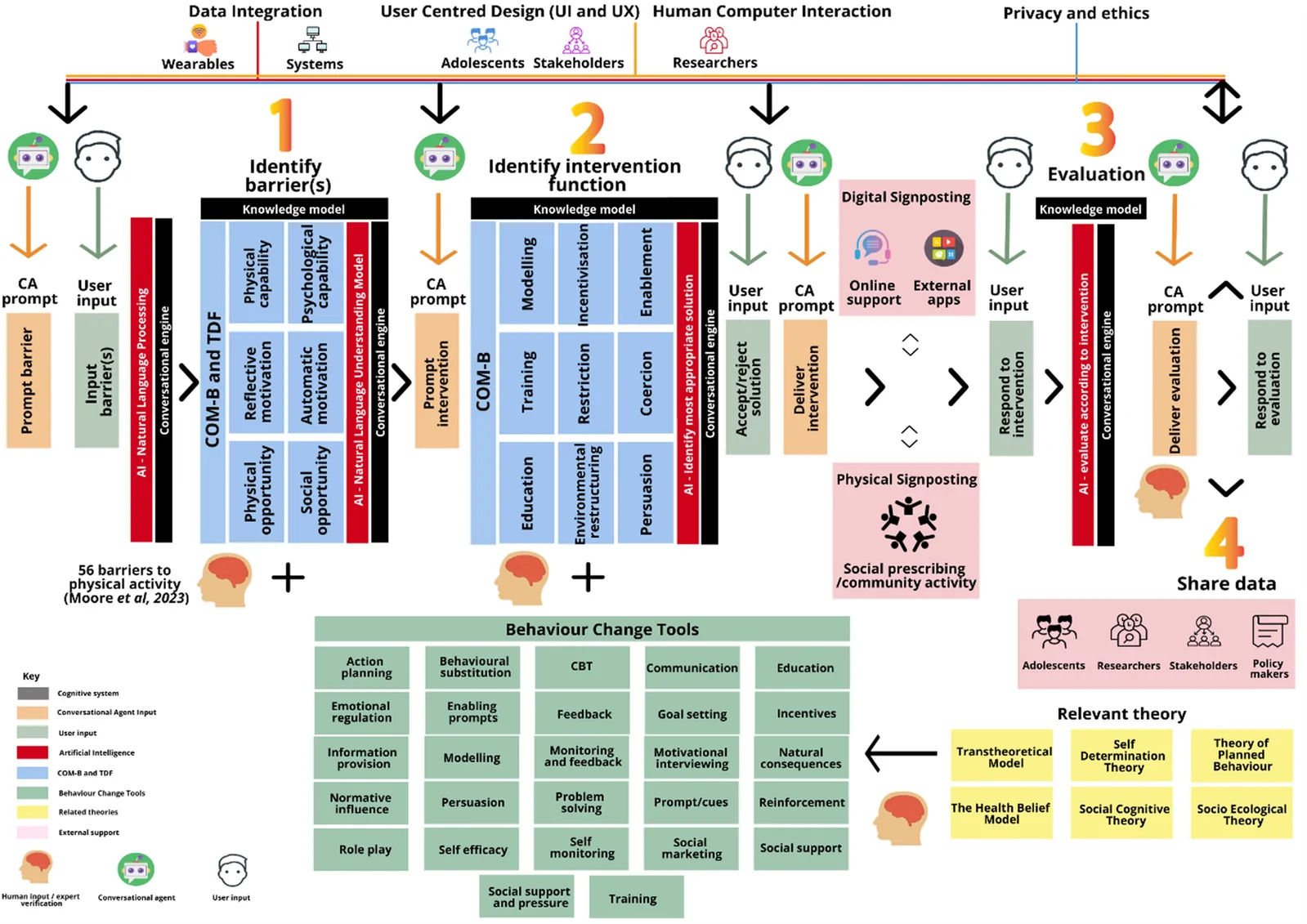
PhysB Framework for overcoming barriers to PA. The framework combines user input to identify barriers, deliver targeted interventions, and evaluate outcomes using behaviour change theory and expert validation

The purpose of the PhysB framework (Fig. 1) is to inform the design and development of CAs that support adolescents in overcoming barriers to physical activity. It ensures that these agents are grounded in theory and evidence, and that they can accurately identify and categorise barriers from adolescents’ natural language input. It is a novel approach built upon a comprehensive analysis of existing literature, empirical evidence, and theoretical perspectives employing philosophical, theoretical, and technical considerations. It also aims to provide a broad framework to support innovative approaches in this field incorporating different ontological approaches. The PhysB framework presents the symbiotic connection between the user process at four phases – identification of barriers (Phase 1), identification of intervention functions (Phase 2), evaluation (Phase 3), and data sharing (Phase 4). It presents the CA’s role in identifying and delivering solutions to barriers to PA encountered by adolescents. It also examines the cognitive system that orchestrates the CA’s dynamic interactions with users. This system comprises a knowledge model equipped with the information needed to offer evidence-based assistance to adolescents. It is coupled with a conversational engine that interfaces with the knowledge model and provides personalised natural language support tailored to each unique barrier.

### Existing frameworks

Previous research has highlighted the absence of a theoretical foundation in PA interventions delivered via CAs [25, 29]. However, a behaviour change model for CAs delivering lifestyle modification programmes has been previously developed. It consists of four components: (1) designing CA characteristics and understanding user background, (2) building relational capacity, (3) building persuasive conversational capacity, and (4) evaluating mechanisms and outcomes [29]. The PhysB framework can be used in conjunction with Zhang’s work [29] as a complementary resource for conversational AI designers aiming to develop solutions for identifying and addressing barriers to PA. However, while Zhang’s model offers a comprehensive and holistic approach to CA design, it does not provide a framework for designing the interventions themselves. These interventions are crucial when aiming to deliver effective, evidence-based, user-led approaches. For example, it is important to determine which interventions should be included in the knowledge model, how they will be designed, and which theories will inform their development. Additionally, the evaluation process should be carefully considered to ensure effective assessment of the interventions’ impact.

Other established frameworks for digital health intervention design and evaluation that have informed the development of the PhysB Framework include well-recognised, evidence-informed models from the digital health and behaviour change literature:

- The Medical Research Council (MRC) Framework for Developing and Evaluating Complex Interventions outlines a non-linear, iterative process comprising development (or adaptation), feasibility/piloting, evaluation, and implementation phases. It stresses repeated consideration at each phase of six core elements identifying key uncertainties, engaging stakeholders, contextual understanding, developing and refining programme theory, refining the intervention, and economic evaluation [30].
- The CONSORT EHEALTH guidelines extend the CONSORT standard to web- and mobile-based health interventions, offering a structured checklist that improves the transparency, quality, and replicability of reporting. This includes detailed elements such as intervention delivery mode, technical infrastructure, usage analytics, and adherence [31].

While both frameworks provide methodological rigour and reporting standards, they do not fully address the unique requirements of CAs for promoting PA among adolescents. Thus, while these frameworks are invaluable for guiding the development and evaluation of digital interventions broadly, they should be supplemented with CA specific design considerations to fully support interventions in school and community settings for PA promotion.

### Theoretical underpinning and system structure

The PhysB framework incorporates key constructs from robust theoretical models and frameworks, such as the Capability, Opportunity and Motivation Behaviour model (COM-B) [32] and Theoretical Domains Framework (TDF) [33] to support Phases 1 and 2. It also draws from existing evidence of behaviour change tools [26] informed by the behaviour change taxonomy [34], to facilitate the design of effective interventions. The framework adopts a cross-sectional approach, guiding the technical or machine-centred ontology to ensure the accuracy, safety, and evidence-based nature of the cognitive system that includes both the knowledge model and the conversational engine. Moreover, the framework provides guidelines for the social or human-centred ontology, promoting design principles that foster engagement and trust. Additionally, it informs evidence-based content design, enabling the CA to interact effectively with adolescents while possessing persuasive and relational capabilities. The following details how existing theory will inform the conceptualisation of the PhysB framework and consequently will inform the ontology and design of CAs in this domain.

### Epistemology

**Fig. 2:**
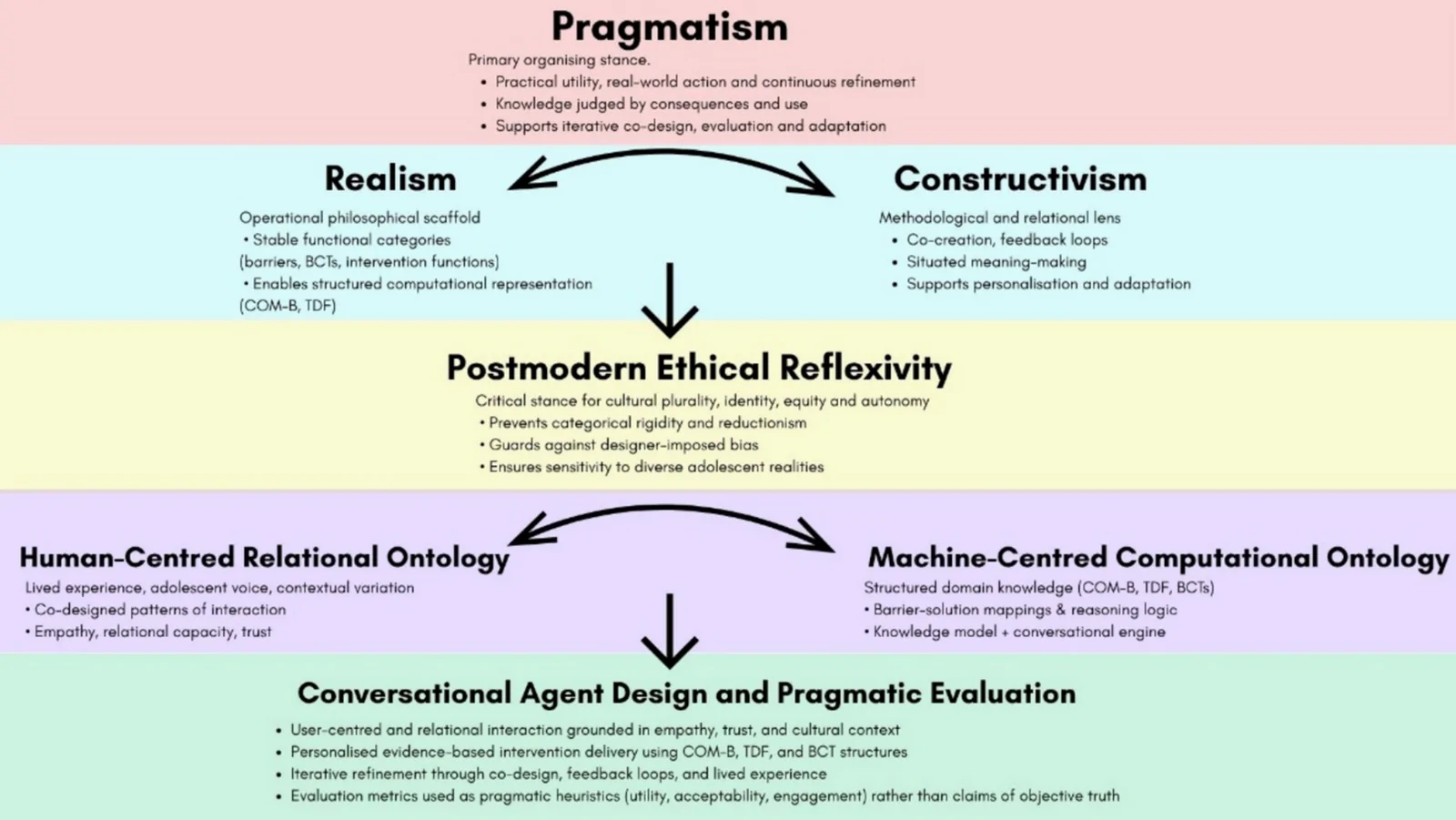
Meta-Theoretical Synthesis Model (PhysB). The model illustrates the integration of pragmatism as the primary epistemological stance, supported by realism and constructivism in operational and methodological roles, with postmodern ethical reflexivity providing critical oversight. This synthesis informs a dual-layer ontology (human-centred relational and machine-centred computational) that shapes the design of personalised, evidence-based, and ethically reflexive CAs, evaluated through pragmatic heuristics of utility and real-world effectiveness

Epistemology underpins the PhysB framework by shaping how knowledge is acquired, interpreted, and evaluated (Fig. 2). While conversational AI touches on broad philosophical domains (e.g., epistemology, ethics, logic), PhysB adopts pragmatism as its primary stance [35]. Pragmatism anchors the framework in practical utility, iterative learning, and real-world effectiveness, valuing knowledge for its ability to support personalised, context-responsive interactions that help adolescents overcome barriers to physical activity.

Complementing pragmatism, realism and constructivism play supporting roles. Realism treats behavioural constructs (e.g., barriers, solutions) as sufficiently stable categories for intervention design and computational modelling, reinforcing the use of established systems such as COM-B and TDF [36].

Constructivism informs how knowledge evolves through co-creation, feedback, and context-dependent meaning, and is embedded in participatory design and iterative evaluation cycles [37]. Together, realism provides structure, and constructivism enables adaptation in service of practical outcomes.

A postmodern perspective contributes as a critical lens by challenging universal categorisation and highlighting the plurality of lived experience [38]. Rather than defining epistemological foundations, it functions as ethical reflexivity, guarding against reductionism and bias when working with diverse adolescents.

Aligned with pragmatism, evaluation metrics such as reliability and generalisability are treated as heuristics for practical utility rather than indicators of objective truth. Knowledge and system behaviour are understood as provisional and open to continuous refinement based on real-world evaluation.

### Ontology

Ontology defines the core entities, concepts, and relationships that structure the PhysB framework’s knowledge model [39, 40]. The PhysB ontology comprises two interconnected layers (Fig. 2):

- Human-centred relational ontology, which grounds the system in adolescents' lived experiences, barriers, motivations, and social context.
- Machine-centred computational ontology, which structures these concepts to support reasoning within the conversational engine.

This dual structure reconciles relational meaning-making with computational needs. From a pragmatist–constructivist perspective, ontologies in PhysB are functional tools that evolve through co-design, pilot implementation, and empirical feedback rather than fixed representations of behavioural reality.

Realism supports structured behavioural constructs within the computational layer, using taxonomies like COM‑B and TDF to classify barriers and align evidence‑based behaviour change techniques [18].

Constructivism shapes the evolution of this structure through adolescents situated experiences, enhancing adaptation and personalisation. Postmodern critique safeguards against rigid categorisation, encouraging sensitivity to values, identity, culture, and autonomy.

The ontology influences both the content and logic of the knowledge model, ensuring intervention strategies are theoretically grounded, ethically responsible, and practically useful. Large language models (LLMs) may operate within this architecture as adaptive processing mechanisms that support interpretation and natural language interaction. They do not define ontological structure; human designers retain accountable authorship within the principled boundaries established by PhysB.

Ultimately, the purpose of the PhysB ontology is to guide personalised, evidence-based conversational interventions that dynamically respond to adolescents’ evolving needs. Its pragmatic orientation supports ongoing evaluation and refinement, positioning PhysB as a conceptually scalable yet empirically provisional framework ready for further operational testing.

### Knowledge model and cognitive system

**Fig. 3:**
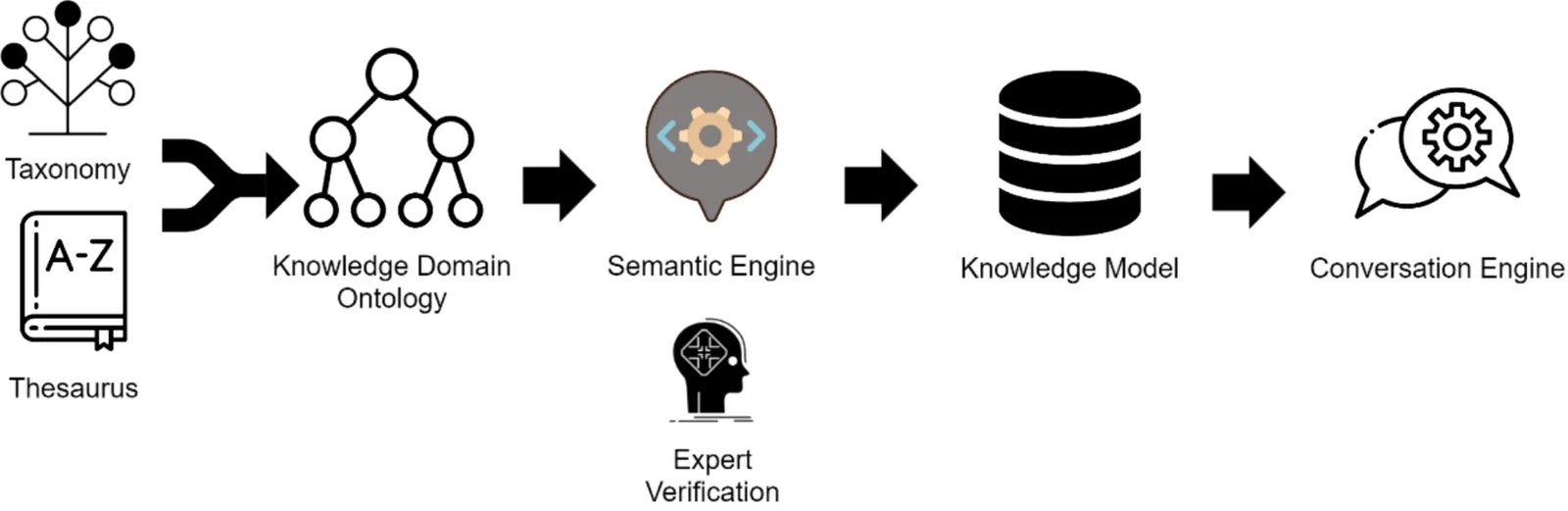
Overview of Ontology-based Conversational AI Agent Design Process This diagram illustrates the structured design pipeline of a conversational AI agent grounded in semantic technologies. Core components such as taxonomy, thesaurus, and domain ontology feed into a semantic engine, which integrates expert-verified knowledge to create a robust knowledge model. This model powers the conversation engine, enabling intelligent, context-aware dialogue generation and user interaction

Within the PhysB framework, the knowledge model forms the structured representation and organisation of information that the cognitive system uses to interpret user input and generate appropriate responses. Ontology guided CAs (Fig. 3) are sophisticated conversational AI systems that employ structured knowledge representations (ontologies) to proficiently comprehend and address user queries, thereby ensuring effective communication. These CAs are primarily designed with the meticulous objective of accurately mapping the extensive knowledge domain they operate within [41]. Through drawing on an ontology that defines the core concepts, barriers, relationships, and behaviour-change strategies relevant to adolescent PA, the CA can map user-reported barriers to theoretically grounded solutions with precision [42].

The development of such systems requires collaboration with domain experts, who diligently identify and gather pertinent taxonomy and resources. These resources are then integrated to form a comprehensive knowledge domain ontology, which is subsequently processed by a semantic engine to generate a structured knowledge model [43]. This process enables the transformation of theoretical and empirical knowledge into operational dialogue patterns, including barrier categories (e.g., psychological capability, opportunity, motivation), associated behaviour-change techniques, and tailored intervention strategies.

The resulting knowledge model underpins the conversational engine, which dynamically selects and constructs contextually relevant responses using knowledge graphs, rule-based reasoning, or neural architectures [42]. Within PhysB, this architecture enables the categorisation of adolescent-reported barriers and identification of evidence-based solutions, supporting personalised and theoretically aligned intervention delivery.

#### Cognitive system

The cognitive system integrates the knowledge model with the conversational engine to interpret user input and deliver theoretically grounded and personalised behaviour-change support to adolescents. Its role is to enable the identification of intervention functions and behaviour change tools that offer personalised support tailored to adolescents. Drawing on established behavioural theories enables the system to deliver theoretically coherent, safe,and eff ective interventions and alignment with user needs.

#### COM-B

The Capability, Opportunity, and Motivation (COM-B) model serves two primary purposes within the cognitive system [32]. Firstly, it facilitates the classification of barriers faced by adolescents into six domains: capability (physical and psychological), motivation (reflective and automatic), and opportunity (physical and social). By doing so, it aids in the identification of sources of behaviour, suggesting that behaviour is influenced by three interacting factors: capability (the individual’s ability to perform the behaviour), opportunity (the availability of resources and environment that support the behaviour), and motivation (the individual’s drive to perform the behaviour) [32]. These barriers are then appropriately categorised within the knowledge model. Secondly, after identifying these barriers, they are further categorised into one of nine intervention functions, such as education, training, or modelling. When coupled with the TDF, this categorisation aids in the selection of suitable behaviour change tools. COM-B therefore complements the pragmatist stance adopted within PhysB, acknowledging the complex and dynamic determinants of behaviour.

#### TDF

The TDF is a comprehensive framework that integrates 128 theoretical constructs from 33 behaviour change theories into 14 domains [33]. In conjunction with the COM-B model, the TDF utilises existing taxonomies or databases of Behaviour Change Techniques (BCTs) to select appropriate techniques corresponding to each domain. It facilitates a deeper understanding of the underlying determinants of the target behaviour. This understanding, when combined with the knowledge model and conversational engine, guides the selection and customisation of specific BCTs that are likely to be effective for the given behaviour and target population. Both the COM-B and TDF can also be used to guide and assess the fidelity and effectiveness of behaviour change interventions. Finally, the TDF is rooted in a range of existing theories such as the Health Belief Model [44], Theory of Planned Behaviour [45], Social Cognitive Theory [46], Self Determination Theory [47], the Transtheoretical Model [48], and the Social Ecological Model [49], all of which are relevant theories in the domain of PA.

#### BCTs

BCTs are discrete, theory-linked intervention components such as goal setting [50], self-monitoring [51] and problem solving [52]. Within the PhysB knowledge model, BCTs are categorised using COM-B and TDF analysis and delivered through the conversational engine. These techniques should be co-designed with adolescents and validated by experts to ensure relevance, engagement, and conversational deliverability. Adoption of the BCT Taxonomy facilitates consistency, comparability, and replicability across CA-delivered interventions and contributes to wider evidence synthesis.

#### Conversational engine

The conversational engine should be user-led and cultivate relational, persuasive, and empathic communication [25]. Incorporating user-centred design principles [53] and established human-computer interaction frameworks [54] ensures usability, acceptability, and safety. This approach supports the social ontology and reflects a constructivist, participatory ethic in which adolescent experience actively shapes system refinement.

### CA design

The delivery of interventions alone may not be sufficient to drive behaviour change unless the conversational engine can effectively engage adolescents and prove to be an acceptable and usable solution for this population. The design of the CA must therefore be grounded in User-Centred Design (UCD), Human-Computer Interaction (HCI), and conversation design principles to ensure interaction is user-led, contextually informed, and capable of sustaining engagement and adherence. This aligns with the social or human centred ontology of PhysB, emphasising relational, ethical, and participatory interaction.

#### Understanding user context

Understanding user context including developmental stage, cultural background, identity, preferences, and lived environment, is essential to tailoring interactions and developing relational capacity. Contextual information supports the personalisation of interventions and allows the CA to adjust guidance according to the individual’s behavioural journey. For example, the Transtheoretical Model (TTM) [48] provides a framework for assessing readiness to change and designing stage-appropriate support. Through monitoring and reflection, the CA can identify a user’s current stage of PA engagement and adapt support dynamically, enhancing intervention relevance and effectiveness.

#### UCD

UCD prioritises the needs, goals, and experiences of end users to design systems that are usable, meaningful, and engaging [53, 55]. Within PhysB, UCD incorporates co-design and co-creation methods that position adolescents as partners in shaping interaction style, tone of voice, and features. This ensures that the CA’s design aligns with adolescents’ preferences and is continuously refined based on experience and real-world use, reflecting the pragmatist and constructivist orientation of the framework.

#### HCI

HCI plays a pivotal role in ensuring the design of the CA also leads to effective and engaging interactions between users and the CA [54]. If applied properly the CA should have a seamless and intuitive interface, employing clear and concise language, which may include visual elements, such as icons, images or videos, to enhance communication and comprehension. It could also incorporate interactive features to enhance the delivery of interventions, such as providing prompts for goal setting [50] or progress tracking, to foster user engagement and motivation. Feedback mechanisms, including positive reinforcement [56] and constructive guidance [57], can be employed to support users in maintaining their PA routines.

Accessibility and inclusivity are also important considerations when applying HCI principles. The CA’s capacity to seamlessly adapt across diverse technological platforms and devices, while ensuring adolescents' convenience, remains a pivotal requirement. Integration of periodic usability testing [58] should also be applied to harmonise with a user-centric design approach that spans the entirety of the design process.

#### Persuasive technology

Persuasive technology draws from theories of psychology and behaviour change to design technologies that motivate and persuade users to adopt desired behaviours [59]. The approach encompasses the theory-driven solutions to barriers, user design and the understanding of user context. This may entail the incorporation of established theories, such as those found in the BCTs taxonomy, along with other approaches like motivational interviewing [60], social support, encouragement, gamification [61], goal monitoring [50], progress tracking, and adaptive feedback [62]. These strategies are aimed at offering personalised support to individuals and influencing behaviour.

Together, these design principles ensure that the CA operates not merely as a channel for information delivery but as a relational, ethically-grounded, and evidence-based partner in behaviour change, aligned with the philosophical, ontological, and cognitive foundations of the PhysB framework.

### Integration of emerging AI technologies and LLMs

The PhysB framework has inherent adaptability to accommodate innovative and evolving technologies, which may enhance both the knowledge model and/or conversational engine. For example, the knowledge model may incorporate knowledge graphs or neural networks, while the conversational engine may operate through intent-based approaches or integrate an LLM. Regardless of implementation, the framework is designed to align with principles of explainable and transparent AI, ensuring visibility of how behaviour-change recommendations are generated and why specific interventions are selected in response to reported barriers.

#### LLMs

LLMs are sophisticated AI models that undergo extensive training on vast amounts of text data, enabling them to acquire knowledge of language patterns, relationships, and semantic understanding. This facilitates the generation of text that closely resembles human expression. LLMs such as GPT (Generative Pre-trained Transformer) and Bidirectional Encoder Representations from Transformers) have the potential to significantly influence the design of CAs, both now and in the future [63–65]. Within this architecture, LLM components operate as adaptive processing tools rather than autonomous epistemic agents. They support the categorisation and interpretation of natural-language barriers but do not determine the underlying ontology, which remains human-designed and pragmatically governed. Their role is therefore implementation-based rather than foundational, preserving human accountability.

A key strength of LLMs is their ability to understand and generate natural language at a high level of complexity and accuracy based on advanced modelling [64]. CAs are therefore able to engage in more sophisticated and nuanced conversations with humans, including detecting emotions, understanding context, and generating more personalised responses. LLMs can be fine-tuned to specific domains, such as PA or domain specific barriers, which can improve their ability to provide relevant and accurate information. Such models can also generate content automatically, which can reduce the need for manual content creation and maintenance [66]. CAs may then scale more easily and cost-effectively, potentially increasing their reach and impact to augment as a tool to support adolescents and reduce global inactivity levels.

There are also several potential drawbacks to using LLMs in the design of CAs for adolescents [65, 67]. One concern is the potential for bias and misinformation in the generated content [63]. LLMs can learn and reproduce biases [67] and inaccuracies present in the data they are trained on [65], which can perpetuate harmful stereotypes and communicate misinformation. There are also concerns about the privacy and security [63] of the personal information shared through CAs, as well as the potential for unintended consequences or harms. For example, adolescent data could be accessed by third parties, without their consent.

There is emerging empirical evidence that extended use of large language model–based chatbots can be associated with psychosocial risks, including increased emotional dependence and problematic use patterns. A longitudinal randomized controlled study found that higher daily usage of AI chatbots correlated with greater loneliness, emotional dependence on the AI, and reduced socialisation with real people, especially at high levels of engagement across modalities and conversational styles [68]. In related work, research on conversational AI use has documented problematic engagement and emotional attachment to social chatbots, with attachment anxiety predicting greater reliance and maladaptive use behaviours [69]. General-purpose models like ChatGPT may offer broad access to information and social interaction, but their lack of domain-specific tuning and safeguards can contribute to distraction, misalignment with users’ needs, and unintentional misguidance in sensitive contexts. The evidence to date suggests that while conversational agents can provide beneficial support, designers and researchers must carefully consider and mitigate these psychosocial risks, particularly among adolescents whose social and emotional development remains ongoing.

### Evaluation

Integral to the PhysB framework is its role in guiding the evaluation (Phase 3) and subsequent optimisation of both the interventions and the effectiveness of the CA. The process and theoretical underpinning of the framework guide the establishment of clear objectives and outcome measures, tailored to each specific intervention. Researchers and intervention designers can assess the impact of interventions on adolescent PA levels, barrier reduction, and other related outcomes specific to each intervention. Evidence-based optimisation, iterative enhancements, and customisation of the CA become key drivers to better address the needs of adolescents and maximise its potential for fostering positive behaviour change. Evaluation and optimisation of the CA requires data from users, driven primarily through conversations between adolescents and the CA. User input can take various forms, such as natural language interactions in the form of textual messages, responses to prompts or questions, expressed preferences, or results of evaluations requiring survey responses.

Measures such as accuracy, reliability, and generalisability are used within PhysB not as claims of objective truth but as heuristics for practical utility, consistent with pragmatist epistemology. Their purpose is to evaluate whether interventions effectively support behaviour change, not to validate universal behavioural approaches.

The framework is designed to advocate a continuous feedback loop, utilising data from user input and may also include additional data from connected devices, secondary sources, and historical data. Collecting data centrally within the CA enables individual data measurement across interventions, facilitating efficacy and adherence evaluation to inform decision-making regarding adaptations or changes to interventions. By analysing natural language interactions related to physical activity, the cognitive system can effectively provide adolescents with data-driven feedback (Phase 3) that raises awareness of barriers and delivers customised, evidence-based support to motivate and encourage engagement in physical activity. Furthermore, data from multiple participants and interventions can be evaluated to identify overall efficacy and adherence levels or to improve intervention design or adjust the type of interventions applied. It also helps identify common barriers, which facilitates tailored support to address them and reveals patterns or clusters of barriers experienced by adolescents.

Data could be shared (Phase 4) to inform organisations working with adolescents about effective interventions and approaches. It can serve to communicate adolescents' progress, share best practices, and highlight the effectiveness or lack thereof of interventions. For instance, schools can access individual-level data to evaluate effectiveness and offer additional support to students. Additionally, they can analyse group data to discern which interventions have yielded the most and least favourable results within their educational institutions. Such results provide organisations with a holistic understanding of the multiple factors influencing barriers to adolescent PA behaviour, to inform further support or policy decisions. It will also help to build an evidence base for the most prevalent barriers and adherence to solutions, empowering adolescents and key stakeholders to make informed choices regarding the adoption of specific interventions.

The framework promotes the use of the CA as a central hub for evaluation, even when it signposts users to external apps or community activities. Both internal and external interventions can be evaluated through the CA, for example by integrating an online survey or embedding evaluation questions within the dialogue. This central hub facilitates the monitoring of adherence and effectiveness, thus enabling adjustments or modifications to the support provided when certain interventions prove ineffective.

### Trust, privacy and safeguarding

For CAs to gain broader traction as a behaviour change tool, they must earn and maintain trust at individual, societal, and organisational levels. Although trust is recognised as a key determinant of technology adoption in health and well-being domains, it remains underexplored in the academic literature, even as commercial research emphasises its importance alongside perceived risks such as privacy concerns [70–72]. When deployed within sensitive contexts like adolescent PA and health, the stakes for privacy breaches and data misuse become significantly elevated, making robust data protection mechanisms and regulatory compliance imperative for safeguarding vulnerable users.

Adolescent users must be assured that CAs always deliver accurate, age-appropriate content that is relevant to their specific context. Co-designed studies highlight that youth prioritise customisable, privacy-first designs, valuing confidentiality, truthfulness, and risk mitigation features in CAs [73]. Adolescents commonly use AI companion apps, and may spend as much time with these agents as with real friends, raising concerns about impaired social development, unrealistic expectations of relationships, and exposure to harmful content.

CAs not only deliver content but also recommend real-world opportunities for engaging in physical activity, such as local places or programmes. Without verification, referred activities may be inappropriate or unsafe. Scripted guardrails designed in collaboration with trusted entities such as schools, youth services, and public health authorities can help ensure that recommendations are safe, accurate, and contextually appropriate. However, this level of tailored oversight is not feasible with generalised models alone.

CAs must operate under transparent, adolescent-centred data governance frameworks:

- Scholars advocate for digital self-determination, asserting users’ rights to govern their own data, including aspects like consent, accuracy, privacy, and the ability to delete or edit shared information. Data governance models like data trusts, cooperatives, or fiduciaries can redistribute power from centralised actors to individuals and communities.
- Industry-wide research shows that trust, privacy, and security perceptions often overlap and should be assessed together; however, very few studies do so comprehensively in the context of conversational AI systems [74].

Overall, ethics, privacy, and trust should be woven into every stage of CA design from persona creation and dialogue modelling to data collection, personalisation, and evaluation. Frameworks must account for adolescent developmental needs, embed human oversight, ensure transparency, and align with legal requirements (e.g., the EU AI Act’s obligations for disclosure and human supervision of AI systems) as well as broader societal norms. The PhysB framework ensures that ethical governance is therefore conceived not as a fixed protective layer, but as a reflexive, participatory system shaped through continuous engagement with adolescents, stakeholders, and contextual lived experience.

### Integrations

Trust, privacy and safeguarding are also vital when considering how CAs integrate with other systems and devices. For example, adolescents may also utilise wearable devices or mobile apps to track their PA, such as step count, distance covered, or calories burned. The PhysB framework highlights the possibility of integrating with these devices or apps to access relevant data and provide personalised feedback based on activity levels and progress. Additionally, CAs can leverage external data sources like public health databases, fitness databases, or research studies to obtain information on PA guidelines, recommended exercises, motivational content, or evidence-based strategies. This data enriches the CA’s knowledge base, enabling the provision of accurate and up-to-date information to adolescents. With time and continued interaction with the CA, a historical record of interactions and progress can be maintained, enabling analysis to identify patterns, trends, and long-term behaviour changes. This analysis may further empower the CA to deliver more personalised and context-aware recommendations.

### Application of PhysB

The PhysB framework presents a conceptually transferable structure that may inform future work across multiple domains related to behaviour change and conversational AI. While the framework has potential relevance for practitioners, policymakers, researchers, and designers involved in developing conversational interventions, its applicability beyond the prototype context remains conceptual rather than empirically established. The framework should therefore be regarded as a guiding structure that may support decision-making, stimulate collaborative research, and offer a basis from which future intervention development and evaluation can be advanced.

Researchers may utilise PhysB as a conceptual scaffold for designing and evaluating conversational interventions or adapting existing behaviour change frameworks to CA-based delivery. Similarly, practitioners and intervention designers may explore its potential to support structured planning, personalisation, and assessment of interventions. Conceptually, the framework contributes to the field by offering a theoretical mechanism through which conversational interventions might address complex barriers to PA and help inform responses to global inactivity challenges; however, its practical effectiveness and external validity remain to be established through further empirical testing across diverse contexts.

Potential areas for future exploration include public health settings, such as schools, where PhysB may provide a theoretical basis for interventions supporting adolescents to overcome barriers to PA, or healthcare contexts developing multi-component interventions to support young people living with hypokinetic conditions. Beyond PA, elements of the framework may be adaptable to other domains requiring identification and response to behavioural barriers, such as education or mental health. However, such applications represent opportunities for future inquiry rather than established capabilities.

It is essential to acknowledge that significant challenges remain in developing evidence-based conversational interventions. While PhysB offers a structured guide, the creation of effective solutions depends on rigorous expert input, co-design, and systematic testing. As few existing interventions have been designed specifically for CA delivery, further research is needed to examine how multiple interacting barriers can be addressed effectively in real-world deployment. Accordingly, PhysB should be understood as a starting point for conceptual development and research rather than a validated implementation framework.

### Signposting

The discussion thus far has focused on interventions and content deliverable directly through the CA. However, certain barriers may fall beyond the system’s capability to address autonomously, requiring solutions that extend outside the conversational environment. Therefore, behaviour change tools and intervention planning should reflect a broad spectrum of barriers that adolescents may experience, acknowledging that not all needs can or should be met solely within the CA. In such cases, signposting to external support may constitute a critical component of effective intervention design.

To accommodate this, the PhysB framework incorporates the potential for referral pathways to human support or complementary digital services. Drawing on the Social Ecological Model [49] the framework adopts an evidence-based approach that recognises the influence of multiple contextual levels, individual, interpersonal, organisational, community, and policy on PA behaviour. The CA may therefore act as a facilitator, helping adolescents navigate and engage with wider support networks when appropriate.

For example, the CA could:

- Connect adolescents to supportive peers or role models to address interpersonal-level influences
- Provide information about local facilities, clubs, or activity programmes to address organisational-level needs
- Collaborate with community organisations to create supportive environments at the community level
- Promote awareness of local or national initiatives and advocate for changes in policy environments influencing PA access

Integrating signposting as part of a broader intervention acknowledges the pragmatic need for multi-component support and reinforces the role of the CA as a relational facilitator, rather than a standalone solution. This approach supports the social or human centred ontology of PhysB and guards against over-reliance on automated systems for complex behavioural challenges.

## Model use in practice: Phyllis case study

The PhysB framework presented in this paper was applied to create a prototype CA called Phyllis, designed to support adolescents in overcoming barriers to PA [6]. Phyllis is a prototype CA that allows for unconstrained natural language input, prompting users to specify the barriers they face in engaging in PA. The CA was collaboratively designed with adolescents, adhering to user-centered design principles and human–computer interaction guidelines. Its development was guided by an existing behaviour change model [29] and informed by the PhysB framework presented.

An NLU model was created, drawing insights from adolescents in a prior study [18], to encompass a wide range of language, including colloquial terms used to describe barriers to PA. Phyllis’ NLU capabilities were honed using Google Dialogflow, enabling it to grasp input and identify specific barriers referenced within the knowledge model, based on a comprehensive set of 56 barriers to PA. The NLU was pre-trained by the research team to improve the accuracy of the model. Barriers to PA were categorised using the COM-B [32] and TDF [33] to shape both the knowledge model and conversational engine including the NLU model. Specific intents were assigned to each barrier with a minimum of 15 manually generated conversational utterances for each intent. To assess the model’s accuracy, a software program called MindBehind was used to input statements related to each barrier and evaluate the training utterances. The CA’s performance was evaluated using an F1 score, resulting in an 80% accuracy. MindBehind was also used to visually represent the CAs conversation on a canvas and to intelligently route conversations based on identified barriers and solutions.

Two specific interventions were co-designed with adolescents and behaviour change experts to address barriers related to a lack of confidence and motivation for PA. The confidence module was designed based on the Theory of Planned Behaviour [45, 51] and incorporated three evidence-based behaviour change tools identified from the behaviour change taxonomy [34] and a previous study [18]. Tools utilised were problem-solving, verbal persuasion about capability, and self-talk. The motivation module incorporated two specific behaviour change techniques: instructions on how to perform the desired behaviour and behavioural rehearsal/practice. Students received guidance on integrating short (15-minute) sessions of PA into their daily routines, making participation achievable for anyone at no cost. The module also addressed the challenges of educational pressure and time constraints, highlighting how PA can positively impact academic achievement. Moreover, students were provided with links to Sport England websites, offering additional guidance on increasing PA levels in different settings, including at home or outdoors.

In the final phase, the CA was distributed to 46 adolescents aged 11–13 for testing. Users were asked to engage in conversations with the CA, identify barriers they faced, and evaluate how the CA handled different barriers. Feedback on participants’ interactions, including positive and negative aspects, suggestions for improvement, and any observed malfunctions was systematically collected. Participants completed an online survey to assess the CA’s acceptability, usability, and perceived outcomes, incorporating recommended evaluation themes and a questionnaire adapted for children to assess usability. No evaluating mechanisms were built into the prototype so this approach was used. Participants found the CA helpful, informative, likeable, and motivating. They appreciated its ability to ask open-ended questions, emphasising the importance of unrestricted conversation, and providing reassurance. Results demonstrated high acceptability and positive user experiences, fostering increased confidence and motivation [6]. Quantitative data analysis revealed positive user experience scores, while qualitative feedback highlighted participants’ appreciation for the CA’s personality, humour, and use of emojis, icons, and GIFs. Valuable feedback was gathered to improve the CA in the short term, such as incorporating fitness activities into conversations, and in the long term, by enabling deeper personalisation and reducing message text. Overall, these results indicate that adolescents had a positive user experience and would be willing to use a CA like Phyllis in the future.

### Generalisability and use in other populations

The PhysB framework offers a conceptually transferable structure for guiding the design of conversational interventions related to behaviour change and conversational AI. In this paper, PhysB was operationalised in a prototype conversational agent (Phyllis), and this prototype was tested for feasibility, usability, and early user experience with adolescent participants. These prototype results demonstrate that PhysB can inform feasible design and implementation practices within a specific context.

However, it is important to distinguish between prototype evaluation and framework validation. While prototype testing supports the practical viability of design elements derived from PhysB, it does not establish the generalisable empirical validity of the framework across populations, settings, outcomes, or implementation modalities. Accordingly, the framework’s broader applicability remains conceptual and provisional.

PhysB should therefore be regarded as a guiding structure that can support researchers, practitioners, and designers in structured planning and decision‑making. It provides a theoretical basis from which future intervention development, systematic evaluation, and adaptation to other domains can be pursued. Empirical validation through diverse real‑world studies across varied settings, population groups, and health or behaviour outcomes, remains a priority for future research.

## Conclusion

In conclusion, this paper highlights PhysB as an innovative, theory‑driven framework for the design of CAs aimed at supporting adolescents to overcome barriers to PA. While conversational AI shows substantial promise in engaging young people around health behaviours, its effectiveness depends on structured, systematic, and evidence‑based design principles. Integrating behaviour change theory with practical design considerations ensures that the PhysB framework provides a valuable guide for identifying key determinants of adolescent physical activity and informing personalised, contextually relevant interventions, particularly in school‑based settings.

Importantly, PhysB offers a novel contribution by linking theoretical constructs directly with actionable design elements for CAs, enabling researchers, practitioners, and policymakers to make informed decisions grounded in both evidence and real‑world application needs. This evidence‑based orientation helps bridge the gap between conceptual models of behaviour change and the operational demands of conversational AI development.

Nevertheless, significant work remains. Future research should empirically validate the framework through iterative implementation, usability testing with target users, and evaluation of behavioural and engagement outcomes in real‑world deployments. Comparative studies that benchmark PhysB against alternative frameworks or design approaches will further clarify its strengths and limitations. Additionally, exploring the framework’s adaptability across diverse health behaviours, settings, and population subgroups will enhance its generalisability and impact.

Ultimately, by developing theoretically grounded and user‑centred design practice, the PhysB framework has the potential to support positive behaviour change and contribute meaningfully to addressing the global challenge of adolescent physical inactivity.

## Data Availability

No datasets were generated or analysed during the current study.

## Appendix 1

Consultation with the following academics and practitioners took place when designing the theoretical framework:

- Professor Alessandro Di Nuovo – Professor of Machine Intelligence
- Professor Lambros Lazuras – Professor of Sport Psychology
- Professor Rob Copeland—Professor of Physical Activity and Health
- Professor Larissa Davies – Professor of Applied Sport Economics
- Dr Abdel-Karim Al Tamimi—Senior Lecturer of Computer Science and Software Engineering
- Dr Lizzie Freeman – Senior Lecturer in Psychology
- Dr Danielle Paddock – Researcher in Behavioural Science

The PhysB framework was also presented to academics and practitioners in the following research clusters and conferences to gain feedback on the theoretical framework:

- Conversational AI Research Cluster – Sheffield Hallam University—Conversational AI Research Cluster (CAIRC) | Sheffield Hallam University (shu.ac.uk)
- Children and Young People Physical Activity and Sport research group (CYPAS)- Sheffield Hallam University—Children and Young People Physical Activity and Sport research group (CYPAS) | Sheffield Hallam University (shu.ac.uk)
- Re-Work Conversational AI Summit (May 2023)—Speakers | Conversational AI Summit | RE•WORK (re-work.co)
- European Chatbot and Conversational AI Summit (2024)—Home—The European Chatbot & Conversational AI Summit

## Abbreviations

PA: Physical activity
CA: Conversational agent
COM-B: Capability, opportunity, and behaviour model
BCT: Behaviour change techniques
LLM: Large language model
NLP: Natural language processing
